# Fabrication of the macro and micro-scale microbial fuel cells to monitor oxalate biodegradation in human urine

**DOI:** 10.1038/s41598-021-93844-y

**Published:** 2021-07-12

**Authors:** Reyhaneh Yousefi, Mohammad Mahdi Mardanpour, Soheila Yaghmaei

**Affiliations:** Department of Chemical and Petroleum Engineering, Sharif University of Technology, Azadi Avenue, P.O. Box, 11365-9465 Tehran, Iran

**Keywords:** Fuel cells, Chemical engineering

## Abstract

This study presented the fabrication of macro and micro-scale microbial fuel cells (MFCs) to generate bioelectricity from oxalate solution and monitor the biodegradation in a micro-scale MFC for the first time. The maximum generated power density of 44.16 W m^−3^ in the micro-scale MFC elucidated its application as a micro-sized power generator for implantable medical devices (IMDs). It is also worthwhile noting that for the macro-scale MFC, the significant amounts of open circuit voltage, oxalate removal, and coulombic efficiency were about 935 mV, 99%, and 44.2%, respectively. These values compared to previously published studies indicate successful oxalate biodegradation in the macro-scale MFC. Regarding critical challenges to determine the substrate concentration in microfluidic outlets, sample collection in a suitable time and online data reporting, an analogy was made between macro and micro-scale MFCs to elicit correlations defining the output current density as the inlet and the outlet oxalate concentration. Another use of the system as an IMD is to be a platform to identify urolithiasis and hyperoxaluria diseases. As a versatile device for power generation and oxalate biodegradation monitoring, the use of facile and cheap materials (< $1.5 per device) and utilization of human excreta are exceptional features of the manufactured micro-scale MFC.

## Introduction

Applications of microbial fuel cells (MFCs) in bioenergy production, hazardous material detection, and wastewater decontamination have remarkably attracted academic attention^1–3^. Since their emergence in 1970, even though humongous work has been carried out in MFCs domain leading to the exponentially increasing scientific output over the years, there have been limitations to harness MFCs as a viable, workable but cost-effective remedy to the current energy and environmental challenges due to its expensiveness, low performance, and challenges to scale-up^4^. With the variety and scope of their applications, it is vital to develop new processes and devices to overcome these challenges.

Therefore, harnessing the unique features of micro-and -nanoscale in various parts of MFCs, such as electrodes’ size and surface modification, intermediate chamber, geometry, etc., has been the initial intervention to develop micro-scale or (microfluidic MFCs)^5,6^. The implementation of MFCs in microfluidic structures introduces remarkable features such as a higher surface area to volume ratio, efficient substrate utilization, shorter start-up and response time, cost-effectiveness, and eventually, a higher power density^4,7^.

Due to these advantages, microscale MFCs have found new applications in diverse areas, particularly being a bioenergy generator for implantable medical devices (IMDs) in the form of bioelectricity^8^ and/or hydrogen as an effective antioxidant^9^. In addition, macro-scale MFCs can be employed as self-powered biosensors to monitor and survey biological and physico-chemical phenomena by interpreting their electrical responses^10,11^. Successful performance of microbial activity monitoring, biochemical oxygen demand (BOD), toxicants, pH, and temperature variations have been reported in previously published results^12,13^. Despite remarkable studies carried out to assess biosensing and biodegradation monitoring applications of macro-scale MFCs, no study has focused on the use of micro-scale MFCs as a biodegradation monitoring device to observe the concentration changes along the cell. The long response time and large size are common challenges of macro-scale MFCs in this area^14^. On the other hand, there are major obstacles to the development of microfluidic MFC-based biodegradation monitoring devices. In the microscale MFCs, the small amount of the sample in the outlet stream (in the microliter domain) results in a slower sampling process which increases the risk of evaporation and gradual variation in the sample concentration due to the presence of suspended microorganisms. As a result of the presented arguments, since measurement instruments require substantially higher outlet flow rates, specific sampling instruments are to be employed to prevent physical and chemical changes in the analyte.

Due to the mentioned capabilities of MFCs for use in IMDs and the presence of different hazardous organic sources in living organs, oxalate can be introduced as a potential substrate. Oxalate is the ionized form of oxalic acid and is recognized as a toxic end-metabolite in the human body, mainly excreted through the kidney. Renal lithiasis, as a widespread disease affecting between 4 and 15% of the population worldwide, is mainly (80% of cases) caused by oxalate accumulation^15^. Hyperoxaluria, known as urinary oxalate excretion of more than 45 mg day^−1^, over time can induce renal inflammation and a subsequent cascade of autoimmune reactions that disturb renal excretion^16^. Science is still illuminating the role of oxalate in many health issues. Recently, a growing body of evidence has linked the disturbance of oxalates homeostasis with many cardiovascular diseases (CVDs), such as coronary heart diseases (CHDs) and stroke^16^. Moreover, research is beginning to show that other conditions, including chronic obstructive pulmonary diseases (COPDs) and asthma^17^, celiac disease^18^, autism^19^, depression^20^, thyroid disease^21^, and more, may be related as well. Hyperoxaluria treatment includes restrictions of dietary oxalates and/or use of therapeutic drugs. No new drugs have been developed for stone prevention since the 80 s when potassium citrate was introduced^22^. Further, due to inadequate and inaccurate information about the oxalate content in foods^23^, urinary oxalate reduction is not recommended^24,25^, and any oxalate diet should be implemented in conjunction with careful monitoring of oxalate concentration.

Experimental methods mostly reported for oxalate determination are based on physicochemical testing procedures (such as gas chromatography—mass spectrometry, high performance liquid chromatography (HPLC), colorimetric, etc.), However, in addition to being highly expensive, time-consuming, and unsuitable for real-time monitoring (due to the requirement of sample pretreatment), these methods are not accessible in rural areas in developing countries. Moreover, information about the bioavailability and biotoxicity of oxalate cannot be provided. Therefore, the bioaccumulation of the toxicants and their impact on living beings during long-term exposures can only be assessed^26^.

The idea of generating bioenergy from oxalates and measuring their concentration during the biodegradation process in a micro-scale MFC reveals a novel horizon to introduce new applications of microfluidic MFCs as platforms for IMDs. This study developed a bioelectrochemical system to elucidate applications of a microfluidic MFC as an efficient bioelectricity generator and a cost-effective biodegradation monitoring device.

Considering the mentioned problems in measuring substrate concentration in the outlet of the micro-scale MFC, an analogy was used between macro and micro-scale MFCs to elicit correlations demonstrating the output current density as inlet and outlet oxalate concentrations. The performance of the MFCs was characterized based on the polarization behavior, organic loading rate, and equivalent electrical responses of both macro and micro-scale MFCs.

## Materials and method

### Assembly of MFCs

**Figure 1:**
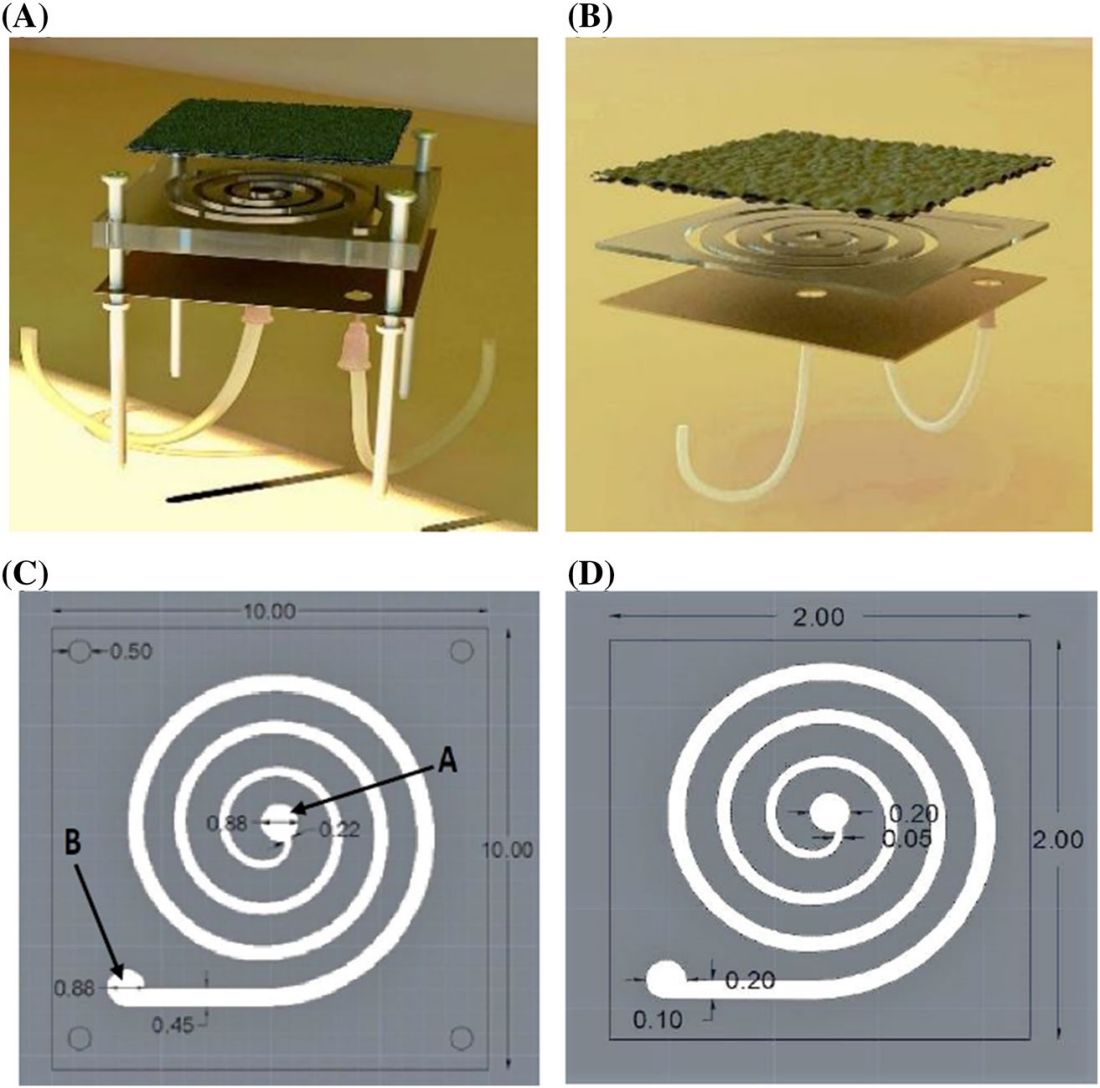
3D schematic images for (**A**) the macro-scale and (**B**) micro-scale MFCs. Images and dimensions of (**C**) the macro-scale and (**D**) micro-scale MFCs. (**A**) and (**B**) show the inlet and outlet, respectively. The scale is millimeter. The used poly (methyl methacrylate) (PMMA) sheets were produced by Cho Chen Industry Co. LTD, Taiwan. The spiral path of the microchannel was drawn by AutoCAD and the file was exported to the laser machine (Non-Metal Laser Cutting Machine, Model CMA1390-LG, GD Han’s Yueming Laser Group Co., LTD). (AutoCAD 2015, URL: https://www.autodesk.com/products/autocad/).

The air-breathing macro-scale MFC is shown in Fig. 1A, which has the exact similar geometry, materials, and architecture to those of the micro-scale MFC shown in Fig. 1B. A spiral channel as the anolyte compartment of the MFCs^9^ was cut on the poly (methyl methacrylate) (PMMA), with a thickness of 1 mm for the micro-scale MFC and 10 mm for the macro-scale MFC using a laser beam. The channel width, fluid passage length, total volume, and finally surface area to volume (SAV) of the macro-scale MFC were 2.2–4.5 mm, 49.64 cm, 18.34 ml, and 7413 cm^2^/cm^3^, respectively (Fig. 1C). These characteristics were 500–1000 µm, 11.03 cm, 84.8 µl and 46,014 cm^2^/cm^3^ for the micro-scale MFC, respectively (Fig. 1D). The used (poly methyl methacrylate) (PMMA) sheets were produced by Cho Chen Industry Co. LTD, Taiwan. The spiral path of the microchannel was drawn by CorelDRAW Graphics Suite 2019, and the file was exported to the laser machine (Non-Metal Laser Cutting Machine, Model CMA1390-LG, GD HAN’S YUEMING LASER GROUP CO., LTD). The cathode, anode, and middle section were attached by an epoxy adhesive glue.

The hydraulic diameter in the spiral channel was gradually increased from 2.2 to 4.5 mm for the macro-scale MFC and from 500 to 1000 μm for the micro-scale MFC. As the substrate flows in the spiral channel, its organic content decreases due to bacterial oxidation. A gradual increase in the hydraulic diameter along the path increased the contact interface between the biofilm and the substrate. It facilitated the mass transfer of the organic materials to the biofilm, which compensated for the shortage of nutrients and prevented power reduction. The centrifugal force in the spiral geometry improves bacterial precipitation and biofilm formation^27^. In addition to the effective role of the spiral channel to increase the flow turbulence, which consequently increases the mass transfer, particularly in the macro-scale MFC, an increment in the passage length compensates for the lack of convective transport between layers because of a laminar flow in the microchannel. It is worth noting that a longer passage length is much more effective than an increase in surface area to volume (SAV) for the mass transfer. For the macro-scale MFC, the dimensions of cathode and anode were 8 cm × 8 cm and 10 cm × 10 cm, respectively. While, for the micro-scale MFC, cathode and anode were installed with the same dimensions of 2 cm × 2 cm.

It should be noted that several macro-scale and micro-scale cells were fabricated, and after an initial assessment (i.e., the open circuit voltage and current density measurement), the best cells were selected to perform the experiments being related to polarization and oxalate concentration monitoring. The difference among the fabricated cells might relate to the quality of catalyst layer coating.

After the identification of suitable MFCs, the assessment of both micro-scale and macro-scale MFCs to obtain polarization curves and variation of oxalate concentrations were done in repeated experiments. For each point, the result indicates the variation of MFCs characteristics among triple experiments. The error bars in the reported figures point out this subject.

### Electrode fabrication

Carbon cloth (ETEK ELAT) was used as the base of the cathode electrode, and the nickel sheet (0.3 mm thickness) was exploited as the anode of the MFCs. The acceptable performance of nickel as a robust, stable, low-cost, high mechanical strength electrode providing a compatible surface for biofilm growth has been proven in previously published studies^8,9^.

**Figure 2:**
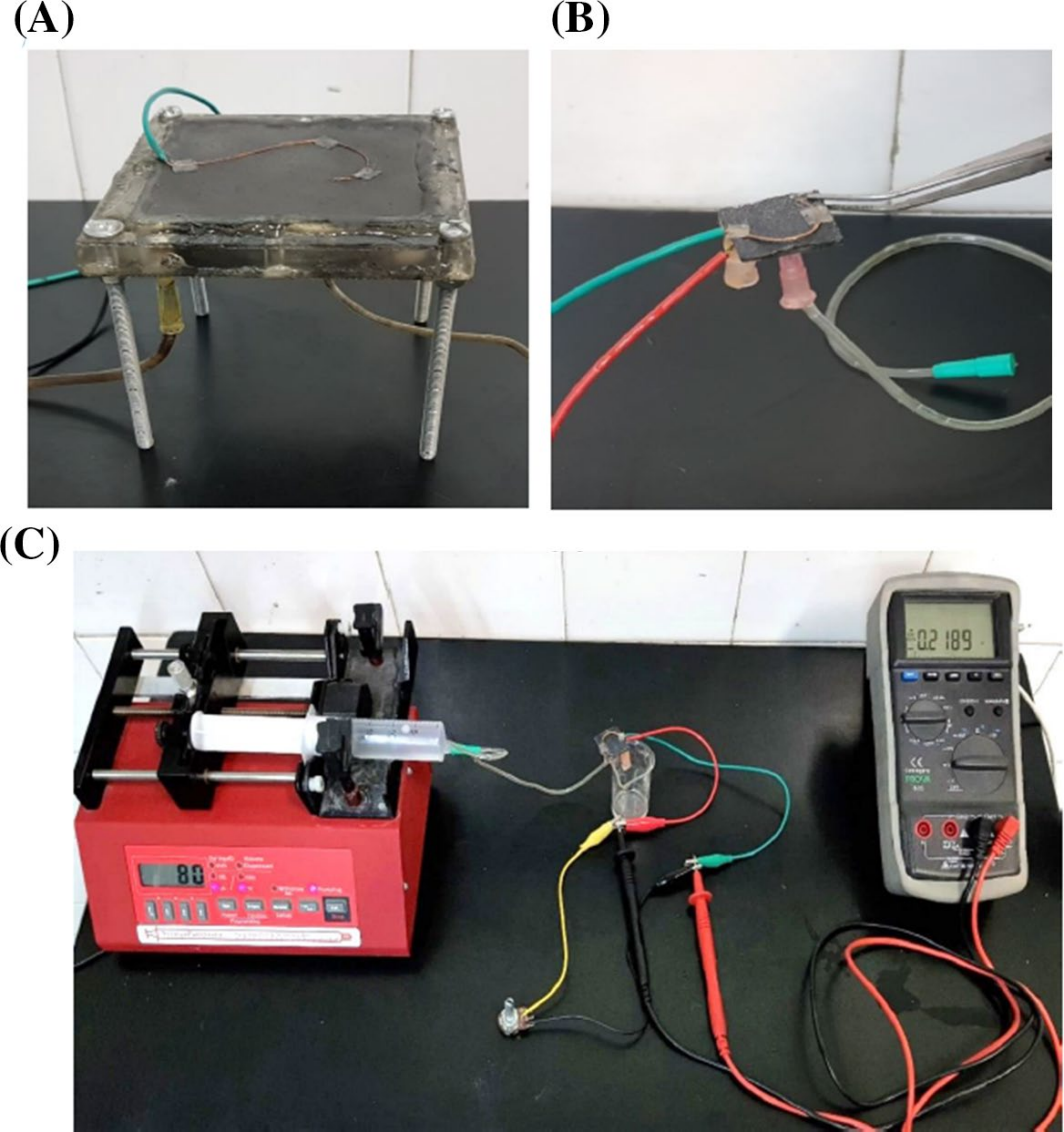
(**A**) the macro-scale and (**B**) micro-scale MFCs, made in this research. (**C**) Measuring the potential difference of the micro-sized MFC in the presence of variable resistance in the circuit.

To conduct the formed water on the air-side of the cathode, a diffusion layer made of polytetrafluoroethylene 60 wt% and carbon Vulcan was covered on the cathode electrodes^28^. To enhance the performance of the MFCs, a catalyst layer was coated on the other side of the cathode. A mixture of carbon/platinum powder (10 wt% Pt/C, Sigma-Aldrich) (0.5 mg Pt/cm^2^ of Carbon Cloth) in conjunction with iso-propanol (33.3 µL/mg of Pt) and Nafion solution (5% Nafion solution, Alfa Aesar) (66.7 µL/mg of Pt) as a high-efficient perm-selective proton exchange membrane was prepared as previously described^28^ and covered on the media-contact side of the cathodes. The Fabricated macro-scale MFC and micro-scale MFC were shown in Fig. 2A,B, respectively.

### Microbial enrichment

Having a uniform and condensed biofilm enriched with electrogens is a sign of an effective microbial enrichment process in bioelectrochemical systems (Zhang et al., 2011b). The microbial enrichment was implemented under open circuit conditions by injecting a mixture of anaerobic sludge (acquired from domestic wastewater treatment unit, Tehran) and substrate solution at a ratio of 1:2 into the MFCs using a syringe pump (New Era; NE-4000; USA). Due to the fact that mixed microbial communities have a wide range of microorganisms, they can consume and decompose a wider range of substrates. The oxalate solution was used as substrate in the MFCs, and the anode was inoculated by anaerobic sludge as a source of electrogens^29^. The normal allowable amount of oxalate in urine (about 156 mg l^−1^)^30,31^ was considered as the substrate concentration for the MFCs. The substrate solution was prepared by solving oxalic acid dihydrate ((COOH)_2_.2H_2_O, CAS 6153-56-6, Merck KGaA) in deionized water. During microbial enrichment, to reinforce the attached bacteria concentration, the concentration of the anaerobic sludge in the feed mixture was gradually decreased until only the oxalate solution was injected to the systems. This issue enhances the priority of the attached bacteria competing with the suspended ones. Therefore, the biofilm will be formed in less time. It is also worth noting that the oxidation of oxalate does not produce noticeable protons; as a result, no buffer will be needed for pH control. The operational conditions such as temperature (298 K), atmospheric pressure (1.044 atm), and other environmental conditions were constant and the same for both MFCs and during all of the procedures.

### Calculations and analysis

#### Electrochemical analysis

The assessment of the open circuit voltage (OCV), produced currentpower densities, and eventually coulombic efficiency were achieved as the electrochemical characteristics. The cell voltage was recorded every 2 min by a multimeter datalogger (PROVA-803, PROVA Instruments Incorporation, Taiwan) connected to a personal computer. Figure 2C shows the total setup. The variation of the OCV was fulfilled during the microbial enrichment of the cells. This indicator gives rise to initial speculation of redox species situations, mass transfer conditions, and an appropriate feed injection rate. To polarize the cells and assess the system overpotentials, the cell circuit was closed to pass the produced current across a variable external resistance in the range of 1 to 690 kΩ. The produced current and power were normalized by the anaerobic anodic compartment volume to compare with previously published works.

In this study, for the macro-scale MFC, the coulombic efficiency was obtained by measuring the produced current and COD removal (Logan et al., 2006). In the micro-scale MFC, due to the sampling problems mentioned in the introduction part, the measurement of COD removal which requires determination of the effluent COD could not be easily achieved. As a result, in this device, the coulombic efficiency was estimated employing the predicted effluent concentrations in part 3.3.

#### Oxalate concentration analysis

The quantitative determination of oxalate concentration was performed using HPLC (High Pressure Liquid Chromatography) in previous studies. But here, another simple and accurate procedure proposed by Yan et al.,^32^ based on the catalytic kinetic spectrophotometric method and by using Victoria blue B (VBB) was used. The outlet stream of the macro-scale MFC was sampled after measuring the maximum produced current density at each substrate flow rate. Each sample was first centrifuged at 3000 rpm, and the supernatant filtered through Whatman No. 1 paper to remove the suspended particles. Since the mentioned method described for measuring concentration is only responsive to a specific range of oxalate concentrations (0.6–9.0 mg l^−1^), the samples were diluted. Two ml of the diluted filtrate were added into a 25-ml volumetric flask containing 1.0 ml of 0.048 mol l^−1^ sulfuric acid solution and 1.0 ml of 0.0012 mol l^−1^ potassium dichromate, and water up to the 20 ml mark. Then, 1.0 ml of 1.00 × 10–4 mol l^−1^ Victoria blue B was added to the flask, diluted to the mark with water, and mixed well. The mixture was immediately placed into a thermostatic water bath for reaction at 60 ° C. After 9 min, the mixture was cooled to quench the reaction with tap water for 2 min. In the next stage, the absorbance values of the final mixture (i.e., A_j_) and the blank solution of the non-catalytic reaction (i.e., A_i_) at 610 nm were recorded by the spectrophotometer (DR 3900, Spectrometer, HACH USA). Finally, oxalate concentration was read from the prepared calibration curve plotted by a series of values of log (A_i_/A_j_) versus oxalate concentration.

## Results and discussion

### Open circuit voltage (OCV)

The biofilm formation under different external resistances was investigated in the work of Zhang et al. It was proved that an increase in the applied external resistance brought about much more uniform biofilm on the electrode surface leads to facilitating substrate diffusion and electricity generation for further experiments. On the other hand, the formed biofilm under the closed-circuit conditions and applying a lower external resistance was not uniform. By applying an external resistance, the attachment of bacteria to the anode surface increases remarkably; as a result, an irregular biofilm will be formed^33^. The shorter lifetime, higher possibility of detachment from anode surface, and difficulty in substrate diffusion into the biofilm are the major challenges of an irregular biofilm. Therefore, the inoculation and electroactive biofilm enrichment for both macro- and micro-scale MFCs have been done under OCV conditions.

To assess oxalate biodegradation conditions through the given potential difference of the redox species and select an appropriate feed injection rate, the OCV variation was monitored for the both macro and micro-scale MFCs by applying different injection rates. The open circuit voltage (OCV) of a MFC can be calculated based on the Nernst equation^34^. In addition to temperature, the activity of the components participating in the redox reaction plays a critical role in OCV variation. Since the activity of components depends on the mass transfer conditions and flow rate highly affects mass transfer conditions, it is crystal clear that during dynamic conditions, the variation of substrate flow rate would change OCV. It is also worthwhile noting that higher flow rates might lead to biofilm detachment and consequently affect substrate biodegradation during the redox reaction. The dependency of OCV of a MFC on the substrate flow rate during dynamic conditions has been reported in previously published results^8^.

At this stage, attempts are made to enrich the biofilm with the least possible discharge to minimize the shear stress applied to the biofilm. On the other hand, the discharge should be large enough to prevent the biofilm from getting dried or separating from the anode surface and the microorganisms from death due to insufficient access to food.

**Figure 3:**
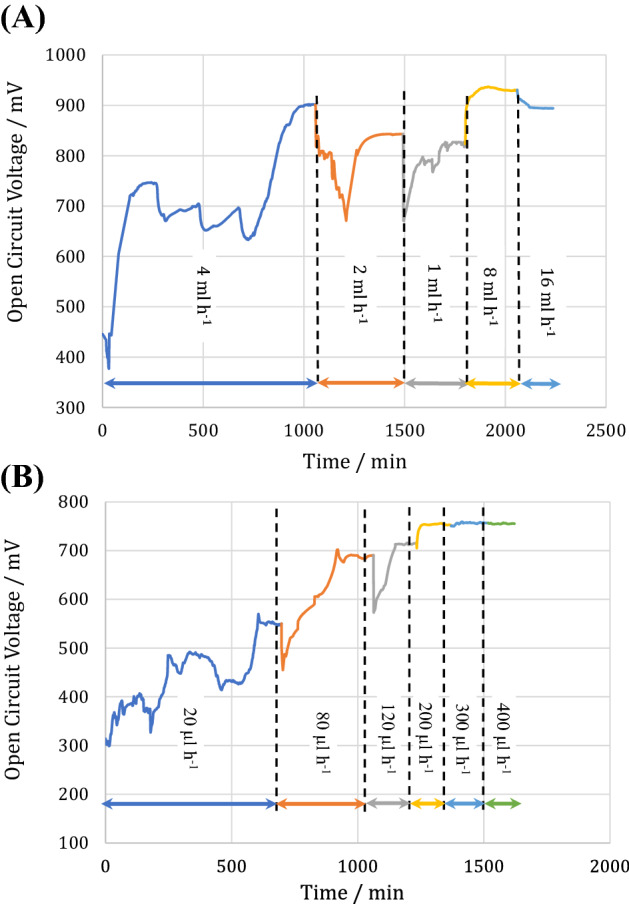
The open circuit voltage (OCV) evolution of (**A**) macro-scale and (**B**) micro-scale MFCs.

For the macro-scale MFC, the feed injection initiated at a flow rate of 4 ml h^−1^ indicating an initial voltage difference of 445 mV and approached 900 mV after 1050 min (Fig. 3A). The OCV increased to 740 mV and revealed the descending trend to 630 mV in conjunction with some fluctuations. During this period, the observed fluctuations might indicate bacterial competition, chemotaxis behaviors among different species, and complex bacterial metabolism during their life.

A decrease in the substrate flow rate to 2 ml h^−1^ led to the abrupt and temporal OCV reduction to 670 mV, which might be attributed to hydrodynamic instability due to the change in the feed injection rate. After a while, the OCV increased to 840 mV. A decrease in the maximum OCV to 2 ml h^−1^ compared to 4 ml h^−1^ implies better mass transfer conditions in higher flow rates. Decreasing the substrate flow rate to 1 ml h^−1^ decreased the maximum OCV to 820 mV indicating the critical role of the feed injection rate on the redox species activities. In order to find the best flow rate which has the maximum OCV, the variation of voltage difference for 2 more feed injection rates was studied.

In the succeeding steps, the OCV monitoring proceeded by increasing the substrate injection rate to 8 ml h^−1^ produced the maximum OCV of 936 mV. The higher anolyte flow rate indicated a sensible decrease in mass transport resistance between the anolyte bulk and the biofilm, making nutrients readily accessible to microorganisms^35^. A further increase in the feed flow rate to 16 ml h^−1^ decreased the OCV to 895 mV suggesting a disturbance in the bioelectrochemical activity of bacteria. This might be due to a decrease in the retention time of the anolyte and/or removal of the attached biofilm with the high shear stress of the anolyte flow^36^.

A similar trend of OCV evolution was obtained for the micro-scale MFC under different substrate flow rates (Fig. 3B). Increasing the feed injection rate from 20 to 200 µl h^−1^ increased the maximum OCV from 550 to 755 mV. However, a further increase in the substrate flow rate to 400 µl h^−1^ did not increase the maximum OCV noticeably. In comparison to our previously published study^8^, reporting the performance of the micro-scale MFC with a single microchannel and similar electrodes, the spiral geometry of a microchannel with a variable hydraulic diameter plays a critical role in increasing the OCV to more than twofold.

It is critical to note that the initial OCV was considerable in the both macro-and micro-scale MFCs (about 437 and 300 mV, respectively). The hydrophilic voltage of nickel to absorb anolyte^37^ and the presence of spiral geometry to reinforce bacteria precipitation^38^ provide a higher OCV and shorter time to anaerobic conditions initiation.

The reduction in the system size reduced the maximum OCV of the cell by 20%, from 935 mV in the macro-scale MFC to 755 mV in the micro-scale MFC. This might be ascribed to the sensitivity of micro-scale MFCs to the oxygen penetration as an undesirable phenomenon, which greatly reduces OCV due to the abduction of electrons produced by bacteria. As one of the features of micro-scale systems is the rapid response to environmental perturbations, such a decrease in OCV with oxygen presence. This illustrates the voltage of a micro-scale MFC to electrochemically sense other materials on a low scale.

### The assessment of the MFCs’ performance by polarization and power density curves

**Figure 4:**
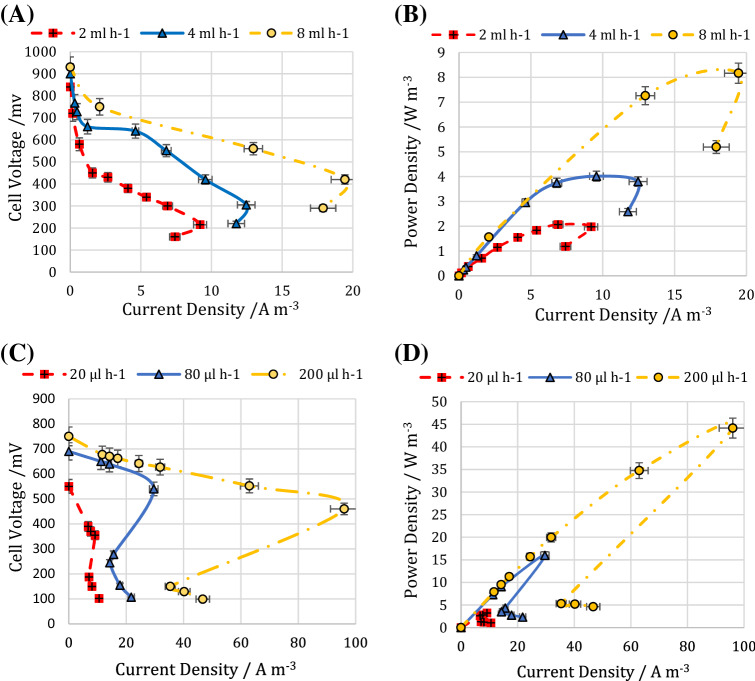
(**A**) polarization and (**B**) power density curves of the macro-scale MFC. (**C**) polarization and (**D**) power density curves of the micro-scale MFC. The error bars represent the variation of power and current densities among repeated experiments.

To assess the performance of the MFCs, a range of external resistances was applied to polarize the cells and reveal their overpotentials (i.e., activation, ohmic, and concentration overpotentials) on the different zones of the polarization curves. The polarization and power density curves were obtained at three different substrate flow rates. As mentioned previously, these flow rates were selected according to the maximum observed OCV during an appropriate period of time. The polarization and power density curves of the macro-scale MFC in the flow rates of 2, 4, and 8 ml h^−1^ are shown in Fig. 4.

The activation overpotentials are considered as the required energy for both substrate oxidation reaction in the anode and oxygen reduction reaction (ORR) in the cathode. The anode activation overpotential chiefly originates from the metabolic energy required or metabolic loss to extract electrons from bacteria^34^. The slop of the initial part of the polarization curve can be used as a qualitative tool to compare the metabolic loss for electron extraction, or in other words, the activation overpotentials of different MFCs^39^. A higher slop of the initial part of the polarization curve indicates a higher voltage drop and implying a higher activation overpotential. In both macro-and micro-scale MFCs, an increase in the substrate flow rate decreased the voltage drop (Fig. 4A,C).

Since the substrate flow rate is the most influential factor in mass transfer resistance in the anolyte, a higher substrate flow rate improves nutrients flow to the biofilm and accelerates its growth. This means more bacteria can oxidate substrate and produce sufficient electrons to compensate for the metabolic loss for electrons extraction. As a result, a higher substrate flow rate decreases metabolic loss by engaging more bacteria in the electron production process and eventually brings about a lower activation overpotential^40^.

Although an increase in the substrate flow rate can increase shear stress on bacteria, causing biofilm detachment, the spiral channel of the MFCs with the variant hydraulic diameter controls shear stress and centrifugal force to improve the mass transfer in anolyte and increase power generation.

Ohmic overpotential which is the linear zone of the polarization curve and implies chiefly the loss of the anolyte electrical resistance revealed a similar slope for the both 2 and 4 ml h^−1^ flow rates. By increasing the feed injection rate to 8 ml h^−1^, the slope of this area decreased. This reflects the role of electron shuttles produced particularly by suspended microorganisms. An increase in the anolyte flow rate might intensify the movement of electron shuttles such as cytochrome c and accelerate electron transfer to the anode, which consequently decreases ohmic overpotential^41^.

The third zone of the polarization curve interprets concentration overpotential or mass transfer overpotential. This zone involves a lower range of external resistances. At low external resistances, the excessive extraction of electrons which cannot be compensated by the redox reactions results in a sudden decrease in both output current and power densities. This phenomenon called overshoot occurs when the demand for electrons exceeds the rate at which bacteria can be supplied^42^. As can be observed from Fig. 4A,B, overshoot occurred in all the substrate flow rates. By increasing the substrate flow rate, the slope of the third part of the polarization or the power density curves increased, indicating a lower reduction in the current density. This means that the higher anolyte flow rate compensates for the depletion of electrons and ions occurring through the overshoot phenomenon. Also, it might assist redox reactions by decreasing the mass transfer resistance^43^.

The maximum produced current and power densities for the macro-scale MFC were 19.44 A m^−3^ and 8.17 W m^−3^, respectively, corresponding to the flow rate of 8 ml h^−1^ and indicating the optimal flow rate of the substrate into the system (Fig. 4B). As can be observed, by increasing the flow rate from 4 to 8 ml h^−1^, the increase in the current and power densities was more intense. This confirms the better performance of the proposed MFC at higher flow rates due to the increase in the organic content as well as improvement in the mass transfer process.

The polarization and power density curves of the micro-scale MFC at different flow rates of 20, 80, and 200 μl h^−1^ are shown in Fig. 4C,D, respectively. The maximum produced current and power densities for the micro-scale MFC were 96.01 A m^−3^ and 44.16 W m^−3^, respectively, corresponding to the flow rate of 200 µl h^−1^. According to Fig. 4C, the voltage drops at the beginning of the polarization curve were not as much as those observed in the macro-scale MFC, as reported in the previous study^44^. Scaling down the system and implementation of a microfluidic structure reduced the amount of dissipation caused by the required activation energy of the cathodic and anodic half-reactions. Also, doing so leads to reducing the energy needed to obtain electrons from organic substrates.

In micro-scale MFCs, the critical role of surface phenomena is undeniable in forming an effective biofilm through an efficient mass transfer of nutrients to the biofilm. Moreover, increasing the substrate flow rate much more intensifies the motivation of the electron’s shuttles in the anolyte compared to that in the macro-scale MFC. These are the main reasons that an increase in the substrate flow rate in the micro-scale MFC resulted in a remarkable decrease in ohmic overpotential (Fig. 4C).

Another feature of micro-scale MFCs is the ability to recover during overshoot occurrence. During recovery, the electron/ion supply and demand balance are reinstated, and the power curve exits the overload mode as the current begins to increase^45^. The recovery highlights the robustness of the microbial culture and its ability to adjust to dynamic and even hostile conditions. This can be observed in Fig. 4C.

The different polarization curves of the two systems demonstrated differences in hydrodynamic conditions, resulting in different resistances, mass transfer processes, and other effects of the microfluidic systems. Even though that the microfluidic system did not achieve the macro-scale OCV, much higher current and power densities and the ability to recover during overshoot occurrence were observed in this system.

It should be noted that scaling down a MFC is always in conjunction with further significant changes particularly in hydrodynamic conditions, organic nutrient accumulation, etc. The main advantages of scaling down a macro-scale MFC to a microfluidic system are providing remarkable control in the hydrodynamic conditions and sufficient nutrient availability as well^46^. In addition, due to the low Reynolds number in microfluidic systems, the laminar flow and a higher inertia force would exceedingly prevent hydrodynamic disturbances. Regarding the channel diameters, injection rates, and substrate characteristics, a laminar flow was established in the spiral channel of the both macro-and micro-scale MFCs. Although it could not be claimed that all hydrodynamic conditions were kept the same during the scaling down, the main characteristics affecting the electrochemical characteristics such as substrate type, inoculation, electrode type, cell geometry, feed injection mode, and ambient temperature were the same. For this step, the difference in the hydrodynamic conditions resulting from the scaling down has been neglected, but the investigation of hydrodynamic conditions in the both macro-and micro-scale MFCs is the subject of future study.

### The assessment of the micro-scale MFC performance as an oxalate biodegradation monitoring device

The most important and innovative goal of this study was to employ a micro-sized MFC as a biodegradation monitoring device which has never been worked before. The small amount of the sample in the micro-scale MFC cause to discredit the analysis of the oxalate concentration in the outlet stream. This is due to the risk of evaporation and gradual variation in the sample’s concentration at the outlet of micro-scale MFC over time due to the presence of microorganisms.

**Figure 5:**
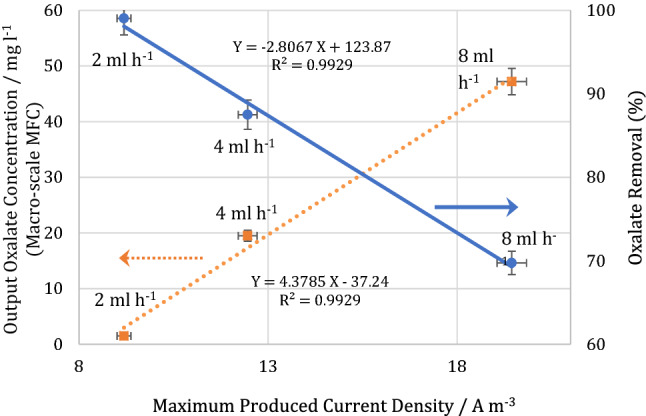
Measured Output oxalate concentration and oxalate removal versus maximum produced current density for macro-scale MFC. The error bars represent the variation of power and current densities among repeated experiments.

In the macro-scale MFC, the oxalate concentration in the outlet stream was analyzed at the maximum point of the power density curve. At this point, the effect of electrical noises was minimum, and the maximum electron generation resulted in the maximum reduction of the organic content. Thus, finding a correlation between the output oxalate concentration and the generated current will be less error-prime. The output oxalate concentration and oxalate removal versus the maximum produced current density at each flow rate for the macro-scale MFC are shown in Fig. 5. The linear behavior of the experimental results revealed an appropriate correlation to obtain the outlet oxalate concentration for the vast range of the substrate flow rates. By measuring the maximum producible current density, the output oxalate concentration was obtained. Regarding the oxalate concentration in the influent, oxalate removal can be acquired. The relative correlation to predict the oxalate removal is also reported in Fig. 5. Moreover, it is worthwhile noting that an increase in the substrate flow rate improved the mass transfer and provided more accessibility of the substrate for the bacteria. This in turn enhanced the maximum produced current density. On the other hand, the increase in the flowrate enhanced the output oxalate concentration and decreased the oxalate removal percentage due to the reduction in the retention time.

To predict the output oxalate concentration and the oxalate removal percentage in the micro-scale MFC, the electrical analogy of the macro-and micro-scale MFCs can be used. The main objective is to obtain a correlation to predict the outlet oxalate concentration in the micro-scale MFC based on the maximum produced current density. The analogy should be implemented so that it can relate the maximum produced current density value in the micro-scale MFC with its equivalent value in the macro-scale MFC. Since a correlation was found between the output oxalate concentration and the maximum produced current density in the macro-scale MFC (Fig. 5), a similar correlation can be acquired for the micro-scale MFC.

**Figure 6:**
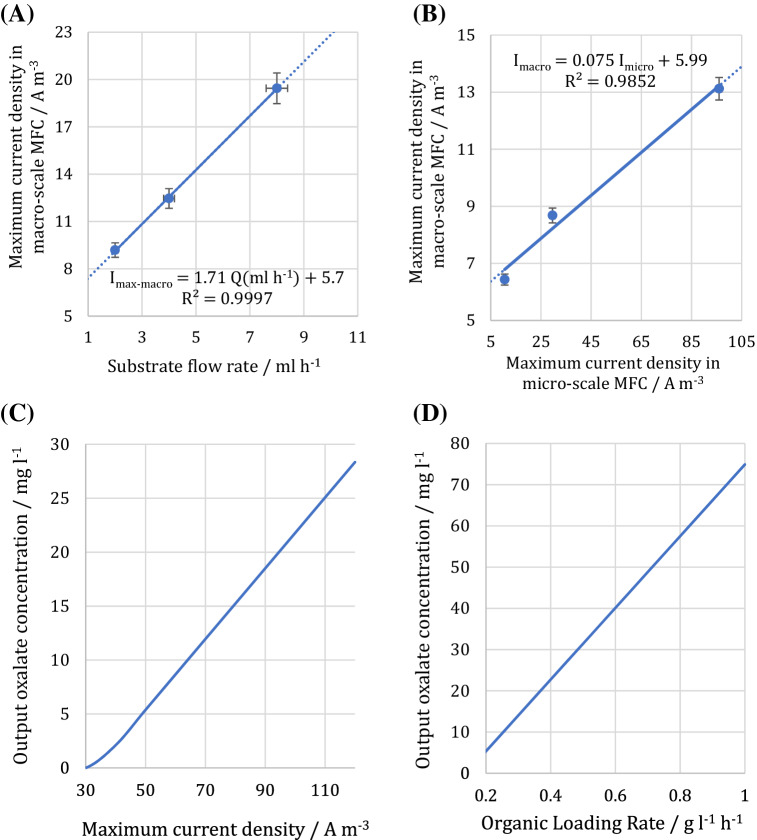
(**A**) The maximum produced current density versus the oxalate flow rate in the macro-scale MFC. (**B**) The variation of maximum current density in the macro-scale MFC versus the related value in the micro-scale MFC. (**C**) The variation of oxalate concentration in the outlet stream of the micro-scale MFC. Oxalate inlet concentration was 156 mg l^−1^. (**D**) Oxalate concentration in outlet stream based on the organic loading rate in the micro-scale MFC. The error bars represent the variation of power and current densities among repeated experiments.

The experimental results reporting the maximum produced current density versus the oxalate flow rate in the macro-scale MFC are presented in Fig. 6A. The presented linear trendline can be used to obtain the maximum current density on the wider range of the flow rate. To make an acceptable analogy between the macro-and micro-scale MFCs, the cell geometry, structure, and materials used for fabrication, operational conditions, and substrate type were the same. Also, due to the low Reynolds numbers in both of the MFCs, the differences in the hydrodynamic conditions resulting from scaling down are negligible. On the other hand, the laminar flow would exceedingly prevent hydrodynamic disturbances, and the mass transfer would mostly occur by diffusion. In this regard, the contact interface between the substrate and the biofilm plays a crucial role in the catalytic reaction and MFCs’ performances. Moreover, the surface phenomena and flow rate considerably influence the mass transport resistances^6^. Hence, the retention time could not be considered as an accurate basis; Instead, the ratio of the surface area to flow rate, depicting the length of time when the substrate is exposed to the biofilm, can be selected as a more viable basis for analogy. Regarding the surface area of the macro-and micro-scale MFCs, the related flow rates of the macro-scale MFC equivalent to the experimental flow rates of the micro-scale MFC were obtained as 0.48, 1.74, and 4.33 ml.h^−1^. With these flow rates, according to the trendline equation presented in Fig. 6A, the maximum current density of the macro-scale MFC was calculated as 6.43, 8.67, and 13.12 A m^−3^. By using these data, the variation of the maximum current density value in the macro-scale MFC corresponding to the related value in the micro-scale MFC is plotted in Fig. 6B.

By inserting the trendline equation presented in Fig. 6B into the correlation of the maximum current density versus the output oxalate concentration (i.e., the trendline presented in Fig. 5), the correlation of output oxalate concentration with maximum current density in the micro-scale MFC was obtained as follows:

1 $$ \left( {Oxalate~\;Outlet\;Concentration} \right)_{{Micro-MFC}} ~\left( {mg~l^{{ - 1}} } \right) = 0.328~~I_{{max}} \left( {A~m^{{ - 3}} } \right) - 11.016 $$

This correlation is plotted in Fig. 6C. Moreover, the maximum produced current density based on the injection flow rate for the micro-scale MFC can be observed in Fig. 4C,D. As a result, a correlation can be obtained between the maximum current density and the flow rate. By combining this correlation with Eq. (1), the outlet oxalate concentration can be acquired based on the injection flow rate. Having written the injection flow rate based on the organic loading rate and considering the substrate inlet concentration, Eq. (2) was denoted as follows:

2 $$ \left( {Oxalate\;Outlet\;Concentration} \right)_{{Micro-MFC}} ~\left( {mg~l^{{ - 1}} } \right) = 86.96~\left( {Organic\;Loading\;Rate} \right)_{{micro}} \left( {g~lit^{{ - 1}} h^{{ - 1}} } \right) - 12.06 $$

This equation predicts the oxalate concentration in the outlet stream for each organic loading rate as plotted in Fig. 6D. Identification of microbial community, separation of dominant species, and employment of genetic engineering to develop a selective oxalate biosensor in a complex organic stream can be the subject of future studies.

**Figure 7:**
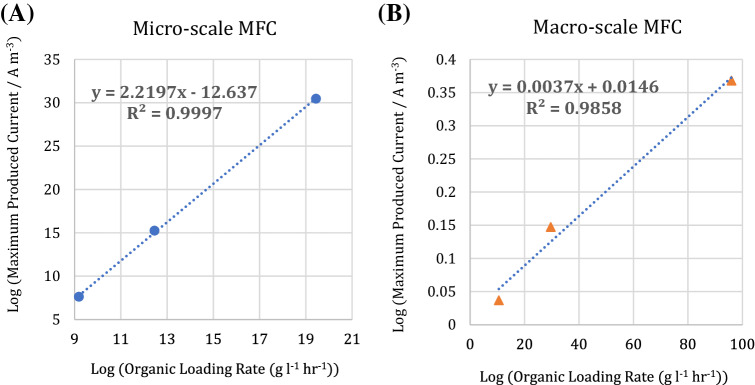
The maximum produced current density versus organic loading rate for (**A**) micro-scale and (**B**) macro-scale MFCs.

Another application of this study could be the use of the micro-scale MFC as a potential biosensor to predict the oxalate concentration in the pure stream of oxalate at the inlet and outlet of a system based on the produced current density. For impure streams, the response of the biosensors can be described as biochemical oxygen demand (BOD). To predict the oxalate concentration in the influent of MFCs, the maximum produced current density was measured at different organic loading rates and correlations corresponding to the experimental results developed. The experimental results and the developed correlations were based on the logarithmic value of organic loading rates and the maximum produced current densities for the both macro-and micro-scale MFCs, as shown in Fig. 7.

### Overall performance assessment of MFCs

The maximum coulombic efficiency of the macro-scale MFC was calculated 44.2% in the flow rate of 2 ml h^−1^. At this point, the oxalate concentration was reduced from 156 to 1.5 mg l^−1^. Based on the output oxalate concentration predicted with the presented correlation, the coulombic efficiency of the micro-scale MFC was estimated to be 25%. Table 1 shows the overall performance of the biosensor MFCs compared to previously published results.

**Table 1:** The comparison of the overall performance of the MFCs.

	Working volume (cm^3^)	Open Circuit Voltage/mV	Maximum current density/A m^−3^	Maximum power density/W m^−3^	Oxalate removal (%)	Coulombic efficiency (%)	Reference
Macro-scale MFC	18.34	935	19.44	8.17	99	44.2	This study
Micro-scale MFC	0.0848	755	96.01	44.16	100	25	This study
Dual Chamber MFC	340	N/A	29.8	N/A	100	21	^47^
Dual Chamber MFC	500	N/A	3	N/A	2	39	^48^

In comparison with previously published results, the macro-scale MFC revealed an acceptable performance in terms of power generation, oxalate removal, and coulombic efficiency. The lower coulombic efficiency of the micro-scale MFC can be attributed to the sensitivity of the system to the oxygen penetration which remarkably decreased the achievable current density.

## Conclusion

**Figure 8:**
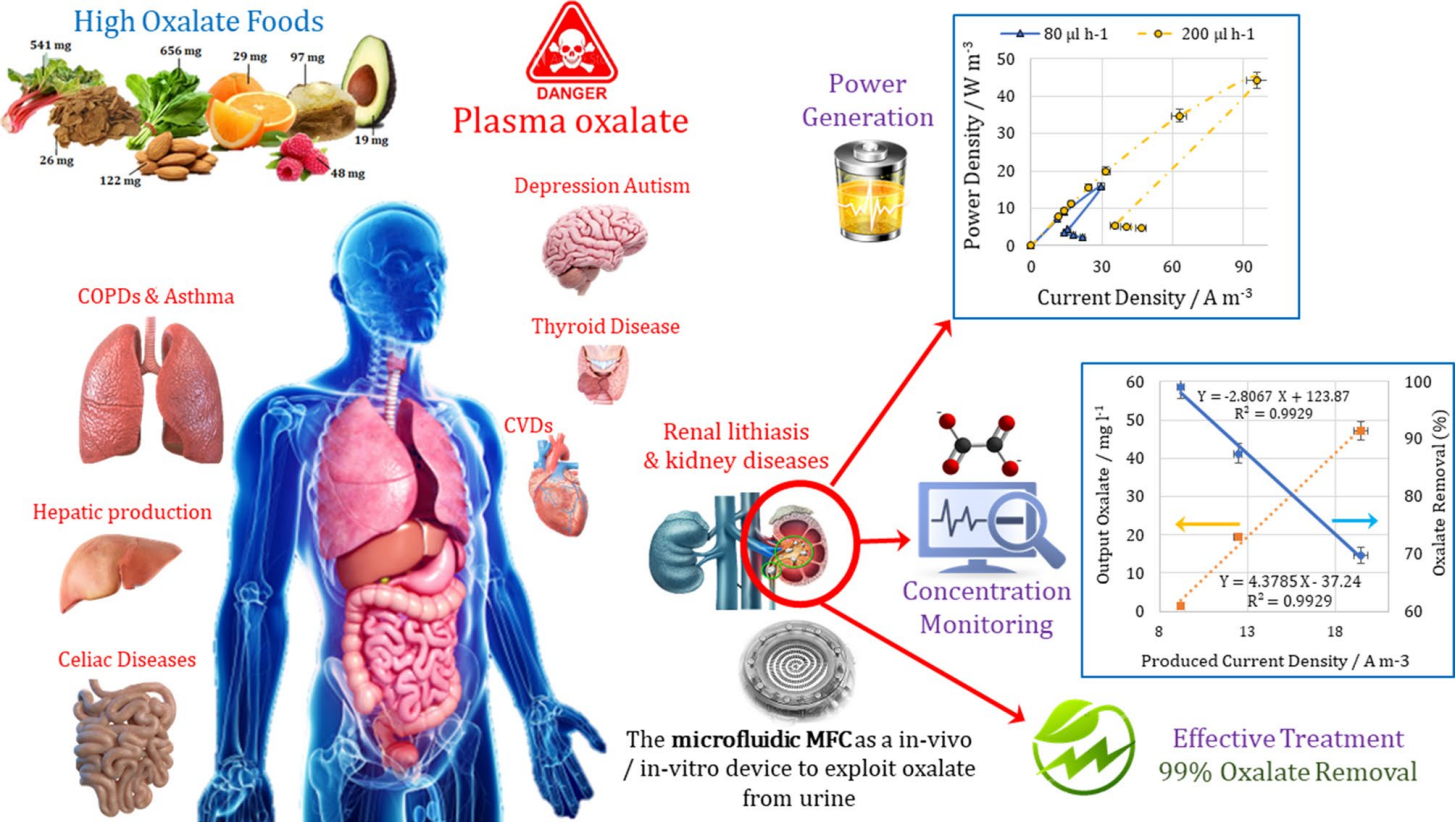
The graphical summary of this study representing the future potential of the microfluidic MFC based biosensor and the obtained results. (The figure created by biorender.com).

The current study demonstrated the ability of the spiral micro-scale MFC to function as a micro-sized biodegradation monitoring device and power generator. The significant OCV of 935 mV in the macro-scale MFC and 755 mV in the micro-scale one, the noticeable generated power, the significant oxalate removal, and finally, the remarkable coulombic efficiency revealed the high potential of the bioelectrochemical degradation process to exploit oxalate as a substrate of MFC. Figure 8 shows the graphical summary of this study.

The stable power generation in the MFCs can be attributed to the presence of the spiral geometry of the anolyte channel. A gradual increase in the hydraulic diameter of the spiral channel from the entrance hole to the exit end compensated for the reduction of the organic content and facilitated bacterial precipitation and biofilm formation due to the centrifugal force. Further, the longer passage length was much more effective in compensating for the lack of convective mass transfer compared to an increase in SAV. In addition, this geometry increased the anolyte flow rate to decrease all cell overpotentials by decreasing the mass transfer resistance, improving the electron shuttle movement, and compensating for electron depletion due to the overshoot phenomenon.

Moreover, the linear correlation obtained between the maximum current density and the outlet concentration in the both macro-and micro-scale MFCs elucidated the electrical analogy and the equivalent experimental results on two different scales. The oxalate concentration in the inlet stream was predicted by a chart and obtained a correlation based on the maximum produced current density.

Here, in order to study the main goal which was to introduce a novel aspect of micro-sized MFCs as a biodegradation monitoring device along with to investigate the biodegradability of oxalate in these systems, anaerobic sludge used as the biocatalyst, which contains a variety of microorganisms. Inoculation with a microbial community helped with the barrier of bacteria and substrate consonant. Employing environmental-and body-friendly and harmless bacteria to biodegrade oxalate are among other topics for future studies. Besides, analyzing the reduction of oxalate amount in the urine or the blood in the in-vivo conditions could suggest as a subject of future researches.
